# Technical Innovation Case Report: Ultrasound-Guided Prolotherapy Injection for Insertional Achilles Calcific Tendinosis

**DOI:** 10.1155/2016/1560161

**Published:** 2016-11-15

**Authors:** Benjamin K. Buchanan, Jesse P. DeLuca, Kyle P. Lammlein

**Affiliations:** Primary Care Sports Medicine Department, Fort Belvoir Community Hospital, Fort Belvoir, VA, USA

## Abstract

We describe the use of ultrasound guidance for hyperosmolar dextrose (prolotherapy) injection of the distal calcaneal tendon specifically just anterior to identified enthesophytes in patients with insertional Achilles calcific tendinosis refractory to conservative treatment. This specific technique has not to our knowledge been described or used in literature previously.

## 1. Introduction

Insertional Achilles tendinosis is a common chronic overuse injury in both athletes and nonathletes alike. Symptoms can last anywhere from weeks to years and cause significant difficulties in daily activities. Treatments can range widely from rest, NSAIDs, topical medications, physical therapy, various injections, and in extreme cases surgical intervention.

Recently, there has been growing interest in prolotherapy injections for this condition [1–3]. Prolotherapy is a relatively safe procedure, with no evidence that points towards decreased tensile strength or increased risk of tendon rupture [2, 4]. One study evaluated radiographic evidence of tendon repair under ultrasonography after prolotherapy treatment that showed reductions in size and severity of hypoechoic regions and intratendinous tears as well as improvements in neovascularity [3]. Evidence is conflicting currently as to the clinical efficacy as well as proper technique of prolotherapy injections for insertional Achilles tendinosis [2]. Thus, further studies are warranted to evaluate the effectiveness of prolotherapy for this condition. This limited technical innovation report is specifically interested in a subset of the previously mentioned patient group who have ultrasound evidence of calcific findings or enthesopathy of the Achilles tendon. The aim of this report is to provide specific ultrasound technique guidance as well as a basis to further evaluate the long term reduction in pain specifically in patients with the diagnosis of insertional Achilles calcific tendinosis.

## 2. Materials and Methods

### 2.1. Ultrasound-Guided Technique

From August 2014 to March 2016, patients presenting to the Primary Care Sports Medicine Department complaining of heel pain, found to have insertional Achilles calcific tendinosis, unresponsive to conservative treatment (i.e., eccentric exercises, Alfredson protocol, rest, and nonsteroidal anti-inflammatory drugs) were treated with an ultrasound-guided prolotherapy technique as described below. Institutional review board approval was obtained for this study and informed consent was obtained from all patients.

#### 2.1.1. Preparation

The patient is placed in the prone position. A towel or pillow is placed under the distal tibia or hung off the side of the table to allow the foot to hang freely in neutral position. The skin overlying the heel should be cleaned and prepared in a sterile manner. We used alcohol based swabs. A nerve block can be performed but most patients do well with a topical spray such as ethyl chloride spray.

#### 2.1.2. Survey Scan

The soft tissues and tendons of the hind foot (Achilles and Plantaris) along with the posterior surface of the calcaneus should be examined in longitudinal and transverse views. A linear or small footprint high frequency (7–12 MHz) ultrasound probe is used. The probe is held on the dorsal aspect of the Achilles tendon over the insertion in long view of the tendon. 1.0 to 2.0 cm depth is all that is needed to visualize areas of the insertion of the Achilles paying particular attention to capturing the entire proximal to distal length of any enthesophyte. Interventions are performed dynamically, with the aid of real-time grey-scale and color Doppler imaging.

#### 2.1.3. Needle Insertion and Injection

**Figure 1:**
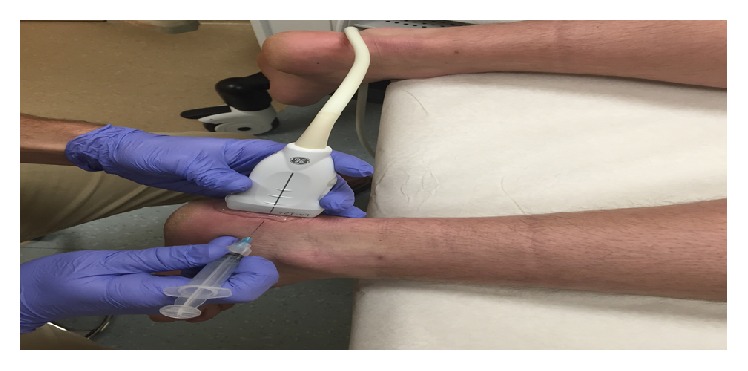
Photograph of sonographically guided prolotherapy injection procedure on 42-year-old male with chronic insertional Achilles calcific tendinosis shows simultaneous use of ultrasound probe and 25-gauge needle to target significant sonographic features.

A 2 mL mixture of 1 mL of 1% lidocaine and 1 mL of 50% dextrose is placed in a syringe. A 25-gauge 1.5 inch standard needle is guided under real-time ultrasound guidance to the insertion of the calcaneal tendon and specifically underneath/anterior to any identified enthesophyte and in very close proximity to calcification. Injections are generally approached from the medial or lateral side in short axis with preference given to shortest injection distance. When the needle tip is clearly visualized at the desired location, 1-2 mL of the solution is slowly injected depending on size of enthesophytic area with 15–20 fenestrations as area becomes anesthetized. Successful targeting of the desired location is confirmed by spread of anechoic fluid within and around the calcaneal tendon just deep/anterior to the enthesophyte (Figure 1, Table 1).

## 3. Results

21 patients with clinically diagnosed insertional Achilles tendinosis were referred to the Sports Medicine Department. Of this group, 10 patients were found to have enthesopathy on ultrasound evaluation. This group included 7 males and 3 females with a mean age of 47 years (range 29–57 years). Achilles pain had been present for a mean of 25 months (range 3–120 months) without significant symptomatic improvement. Ultrasound-guided prolotherapy injection was performed on 5 of the 10 patients.

None of the treated patients had previously received any type of injection therapy. Patients primary pain complaint was with push off. Initially these five patients had an average pain level of 5/10 by visual analog scale (VAS) at rest and 8/10 with sport activity such as running. The Victorian Institute of Sport Assessment-Achilles questionnaire (VISA-A), a previously validated questionnaire used for assessing the index of severity of Achilles tendinopathy, was also used as an objective measure to assess level of improvement. The VISA-A consists of eight questions that measure domains of pain, function in daily living, and sporting activity. Results range from 0 to 100, where 100 represents the perfect score [5]. The average VISA-A score for these patients before prolotherapy injections was 37.

All five patients reported drastic pain relief as well as a return to normal gait and sports activity within 8 weeks of receiving prolotherapy. At that time, the average pain level by VAS at rest was 1/10 with two patients reporting complete pain relief at rest. The average pain level by VAS with sport activity was reported as a 3/10. The average VISA-A score after prolotherapy injections was 84, indicating a sizeable clinical improvement in pain and function. Four patients received an isolated prolotherapy injection. One patient received a second injection 4 weeks after the initial injection. All patients denied recurrence of previous pain levels and symptoms.

## 4. Case Presentation #1

**Figure 2:**
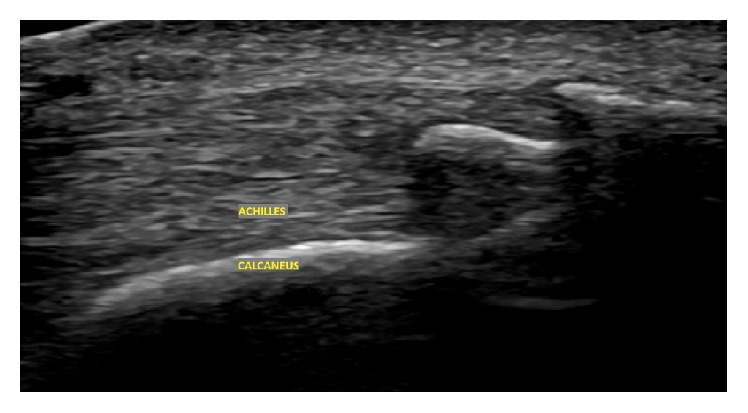
Long axis view ultrasound evaluation in a male with long-time chronic symptoms shows enthesophyte irregularity of the cortex of the calcaneus as well as intratendinous calcification at the Achilles tendon insertion.

A 42-year-old healthy male presented to the Sports Medicine Department with complaint of left Achilles pain for 10 years. He complained of 5/10 sharp pain when walking and 10/10 with running. The pain significantly limited various activities of daily function. His VISA-A score at that time was 35. The patient had been doing physical therapy in conjunction with eccentric home exercises 5 days per week for the previous 8 weeks. He had also tried activity modification as well as nonsteroidal anti-inflammatory drugs with minimal relief. Plain films of the heel were unremarkable but MRI revealed a longitudinal partial tear of the central fibers of the distal Achilles tendon with chronic inflammatory changes. Ultrasound evaluation in the clinic showed a prominent enthesophyte along with insertional mixed echogenicity of the Achilles tendon. The patient continued physical therapy after prolotherapy injection and within a few weeks the patient reported drastic improvement in pain levels. After two months, he reported near complete resolution of pain with levels decreased to 1/10 at rest and 3/10 with running. His follow-up postprolotherapy VISA-A score was 82 (Figure 2).

## 5. Case Presentation #2

**Figure 3:**
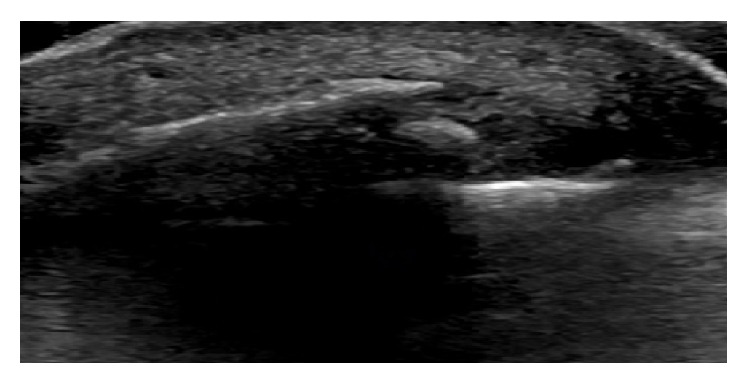
Axial view ultrasound evaluation in a patient with subacute symptoms also shows a large enthesophyte at the insertion of the Achilles tendon.

A 49-year-old female presented to the Sports Medicine Department with the complaint of right Achilles pain for the past 5 months. The patient was an avid runner and reported running about 50 kilometers per week. She reported 4/10 sharp pain when walking and 7/10 with running. Her VISA-A score at that time was 42. She failed conservative therapy that included activity modification, physical therapy, and eccentric exercises, as well as nonsteroidal anti-inflammatory drugs without relief. Plain films of the heel were unremarkable. Ultrasound evaluation showed a prominent enthesophyte in proximity to the insertion of the Achilles tendon. Ultrasound also revealed heterogeneity throughout the Achilles tendon. Similar to the patient in case presentation #1, the patient continued physical therapy after injection and within a few weeks the patient reported drastic improvement. After a couple of months, she reported near complete resolution of pain, with no pain at rest and 1/10 with running. Her follow-up postprolotherapy VISA-A score was 95 (Figure 3).

## 6. Discussion

Prolotherapy is increasingly being used as treatment for a variety of musculoskeletal disorders such as lower back pain [6], knee instability secondary to anterior cruciate ligament injury [7], and osteoarthritis [8], as well as ankle sprains and meniscal injuries [9]. Specifically, in relation to our cases, dextrose prolotherapy seems to show significant clinical efficacy according to case series data for Achilles insertional tendinopathy [1]. Prolotherapy is understood to elicit a proliferant cellular response by inducing inflammation, subsequent growth factor production leading to increased fibroblast proliferation (either locally or systemic), and increased production of extracellular matrix materials [10]. A possible explanation for the success in our cases of enthesopathy is that over time the strongest portion of the tendon was able to “win out” over the bone while being under tensile strength. As the enthesophyte grew, the already weaker tendon insertion deep to the enthesophyte was subjected to a less nourishing environment and began terminal breakdown.

Limitation to procedure efficacy is the fact that sometimes prolotherapy is performed using palpation to guide needle placement. Without visualization, there is higher probability of nonoptimal placement of injectable fluid. Ultrasound guidance will not only aid in precise needle placement but also identify which patients are better candidates for injection as demonstrated by visual evidence of calcification.

Insertional Achilles tendinopathy will often show sonographic evidence of hypoechoicity, intratendinous tears, and increased tendon size. Some subsets of these patients will show calcification at the insertion and/or evidence of enthesopathy. The patients we have treated for this condition fall into this subset category and expand on positive outcomes reported previously [3]. The difference in these cases is that the patients had pain primarily with push off at the area of the enthesophyte and that care was taken to only and specifically inject the small area of tissue between the enthesophyte and the calcaneus. We see potential benefit in both subacute and chronic cases as evidenced by the two previous case presentations.

One limitation to our study given the small sample size is the question of how often prolotherapy injections are needed. Preliminarily, it seems plausible that one injection alone may be adequate given four of our patients only needed one injection. It is however conceivable that some patients with severe refractory cases maybe need additional injections. An additional limitation of our study is that follow-up ultrasound evaluation after clinical improvement was not performed. It would be interesting to see if there was any improvement or regression of calcified areas given the reported improvement in symptoms. It is a possible consideration that the clinical improvement observed is confounded by c-existing pathology of the Achilles tendon as opposed to enthesopathy alone. Previous studies have shown improved sonographic appearance of the tendon after prolotherapy to include neovascularization as well as reduction in tendon size and severity of hypoechoic regions. Regardless of exact etiology of insertional Achilles tendon pain, patients clinically improve with administration of prolotherapy, but more extensive studies and clinical trials are warranted to potentially isolate calcific causes and evaluate the efficacy of our technical approach aimed at injections specifically underneath the enthesopathy.

The numbers are small but the reported outcomes in these patients are encouraging. Ultrasound-guided prolotherapy injection seems to be most beneficial where conservative therapy has failed and there is evidence of enthesopathy or calcific findings and the technique is done as specified. Ultrasound guidance should be the technique of choice for prolotherapy injection for this condition as it provides the highest likelihood that the injectable fluid is placed in deep to the enthesophyte or calcified area.

## Figures and Tables

**Table 1:** Ultrasound-guided prolotherapy injection of insertional Achilles calcific tendinosis in the primary care setting.

Indications	Painful calcific tendinosis unresponsive to conservative therapy
Ultrasound-guided technique	A linear or small footprint high frequency (7–12 MHz) ultrasound probe is used to visualize any calcified area or enthesopathy and target the area just inferiorly for injection
Positioning	Patient is placed in prone position with a towel or pillow under the distal tibia while the foot hangs freely
Needle approach	In plane or out plane of plane approach with a 25-gauge 1.5 inch standard needle
Important anatomy	Achilles tendon, calcaneus, enthesophyte, and Plantaris tendon
Potential complications	Significant complications have not been reported. Possible complications that are typical of similar injections are pain, bleeding, infection, and local irritation
